# Systematic review of human immunodeficiency virus (HIV) knowledge measurement instruments used on the Arabian Peninsula

**DOI:** 10.1186/s13104-015-1614-x

**Published:** 2015-11-04

**Authors:** Maram T. Alghabashi, Barbara Guthrie

**Affiliations:** Bouve College of Health Sciences, Northeastern University, Boston, MA USA; Umm al-Qura University, Mecca, Saudi Arabia

**Keywords:** Saudi Arabia, HIV, Acquired immunodeficiency syndrome, Questionnaires, Health surveys

## Abstract

**Background:**

In 1984, the Kingdom of Saudi Arabia (KSA) began surveillance for human immunodeficiency virus (HIV) incidence and prevalence. However, no culturally-appropriate standardized questionnaire has been developed to measure HIV prevention knowledge in this population. Evidence exists that married Saudi women are especially at higher risk for infection, but lack knowledge of HIV modes of transmission and underestimate their personal risk of becoming infected. The objective of this paper is to present a critical review of existing HIV knowledge measurement tools developed for the KSA and other Arabian Peninsula populations, and to utilize this review to guide the development of a culturally- and gender-sensitive tool. Studies included were in English reporting results of a quantitative survey instrument as either an interview or self-reported questionnaire with questions about knowledge of HIV or AIDS. Surveys must have been given in English or Arabic, and must have been done in a population in the KSA or the Arabian Peninsula. The following data sources were searched for eligible studies: Google Scholar, Google Web, PubMed, PLoS, WHO publications, UN publications, news, and other peer-reviewed publication databases.

**Results:**

Sixteen articles met criteria, and of these, 10 (63 %) were conducted in a KSA population, and a majority of the articles studied students of primary, secondary, or post-secondary schools (n = 9, 56 %). Five studies included only men, while the other 11 included both sexes.

**Conclusions:**

The KSA’s public health goals should more specifically focus on measuring and improving knowledge in high-risk populations such as married women—an option currently limited by commonly available measurement instruments.

**Electronic supplementary material:**

The online version of this article (doi:10.1186/s13104-015-1614-x) contains supplementary material, which is available to authorized users.

## Background

In October 1991, the Saudi Minister of Education directed all educational regions and school health units to implement AIDS education in secondary schools [1]. Until this point, the level of public knowledge about acquired immune deficiency syndrome (AIDS) was generally not addressed in the Kingdom of Saudi Arabia (KSA) for several reasons. At the time, AIDS was an emerging disease with a relatively low prevalence in the KSA compared to other countries, and public discussion of it was taboo [1]. However, beginning with the World AIDS Day in December 1992, measuring the KSA public’s knowledge of AIDS (and later human immunodeficiency virus (HIV) became more important to the KSA government, spurring the development of instruments to measure AIDS and HIV knowledge in addition to related topics, such as attitudes toward infected individuals [2].

When reviewing instruments for measuring HIV knowledge in the KSA, it is important to first consider the writing of Dr. Tariq Madani, a leader in KSA’s Ministry of Health on many infectious disease committees [3]. In his 2004 and 2006 reports on HIV prevalence and the ensuing public health responses in the KSA, Madani emphasized the fact that, “Some of the sexually transmitted infection (STI) preventive strategies that are advocated and used in non-Islamic countries are not acceptable in Islamic countries” [4], implying that prevention strategies in the KSA must be approached differently. “Safe sex” education and needle exchange programs, while useful in other countries, would be unacceptable in the KSA [5].

Further, evidence suggests that the high-risk subpopulations targeted by these preventive interventions are appropriate for study in other countries [6–8], but constitute low-risk subpopulations for HIV in KSA. Studies of sexually transmitted infections (STIs) transmission in the ethnic Saudi Arabian population have identified two main modes of transmission: Saudi men acquiring STIs (including HIV) from sex workers (likely from travel outside the country), and Saudi women contracting STIs (including HIV) from their husbands [9, 10]. As evidence to this fact, Alrajhi reported in 2006 that the main reason given by men for seeking HIV testing at a hospital in Riyadh was due to the experience of symptoms, while “for women, the most common reason was screening because of contact with an HIV–infected spouse, in 73 out of 134 women (55 %)” [11].

Sex workers are staunchly forbidden in KSA [12], so KSA women typically do not contract HIV nor transmit HIV due to sex work. In addition, KSA men do not have the opportunity to hire sex workers unless they travel outside the country. Therefore, it is not surprising that the highest prevalence of HIV among ethnic Saudis appears to be among non-drug using heterosexuals [9, 10], but studies of attitudes in this population reveal a general lack of understanding of modes of transmission [13], and a high level of stigmatization toward those who are infected [14]. Therefore, the methods developed to measure the KSA public’s understanding of and attitudes toward HIV and AIDS should be consistent with both the Islamic faith as well as epidemiologic evidence that would suggest a change in behavior in high-risk subpopulations as a preventive approach.

Evidence exists that women, especially married women, represent a high-risk group for contracting HIV in KSA, but that accurate measurements of HIV incidence and prevalence in this group are lacking, as well as accurate measurements of levels of HIV knowledge. In a study where “data of 5377 reported cases of STIs from all regions of the kingdom during the year 2009 were collected”, 92.9 % of cases were women, almost 60 % of cases were aged 30 and above, and 91 % of cases were married [9]. This suggests that STI knowledge questionnaires used in KSA should be culturally competent so as to accurately measure STI-related knowledge in married women over age 30. A study of yearly reportable case notification rates of HIV in KSA found that among Saudis, women made up between 10 and 20 % of yearly cases 2000–2009 [15].

However, the level of knowledge these women have as to their rights in Islam to various courses of action (such as confronting the husband to go to the clinic, going to the clinic herself, or asking for marriage counselling) is not known. This is because current instruments measuring STI-related knowledge have not typically included KSA women in their cohorts (as will be described later in this paper) [1, 2, 14, 16–18], or if they have, they have used the same identical instrument as used for men [13, 19–29]. Men would require different STI-related knowledge (such as being able to identify symptoms he may have of STIs, or risk behaviors he should avoid with sex workers), so having a unisex measurement instrument for STI-related knowledge in this group would not be culturally competent and would likely not produce a useful or accurate measure of knowledge.

To design KSA’s public health response to the high risk married women apparently face for contracting STIs from their husbands to be well-informed, it is necessary that KSA’s Ministry of Health has accurate information about what these women know and do not know about prevention measures, as well as actions to take if an infection is suspected. It is unclear if questions asking for levels of this type of knowledge are present on existing STI-knowledge questionnaires used in the KSA. The quality of STI-related knowledge measurement instruments that target married women in KSA is not known, as no review of instruments focused on this particular cultural group could be found in the peer-reviewed literature.

With this consideration, the existing literature on measuring knowledge about AIDS and HIV will be reviewed, with a focus on instruments specifically applicable to women in the KSA population. The purpose of this article is to (1) enumerate culturally-competent HIV/AIDS knowledge questionnaires that could inform a current study of HIV/AIDS knowledge in the KSA population (especially women), and (2) analyze them for their strengths and weaknesses in terms of reliability, validity, and cultural compatibility. Specifically, the cultural aspect that will be reviewed is whether or not the instrument selected was used with a culturally-appropriate population. These surveys are reviewed here in terms of their quality and utility in measuring HIV/AIDS knowledge amongst specifically the KSA population.

## Results and discussion

**Fig. 1:**
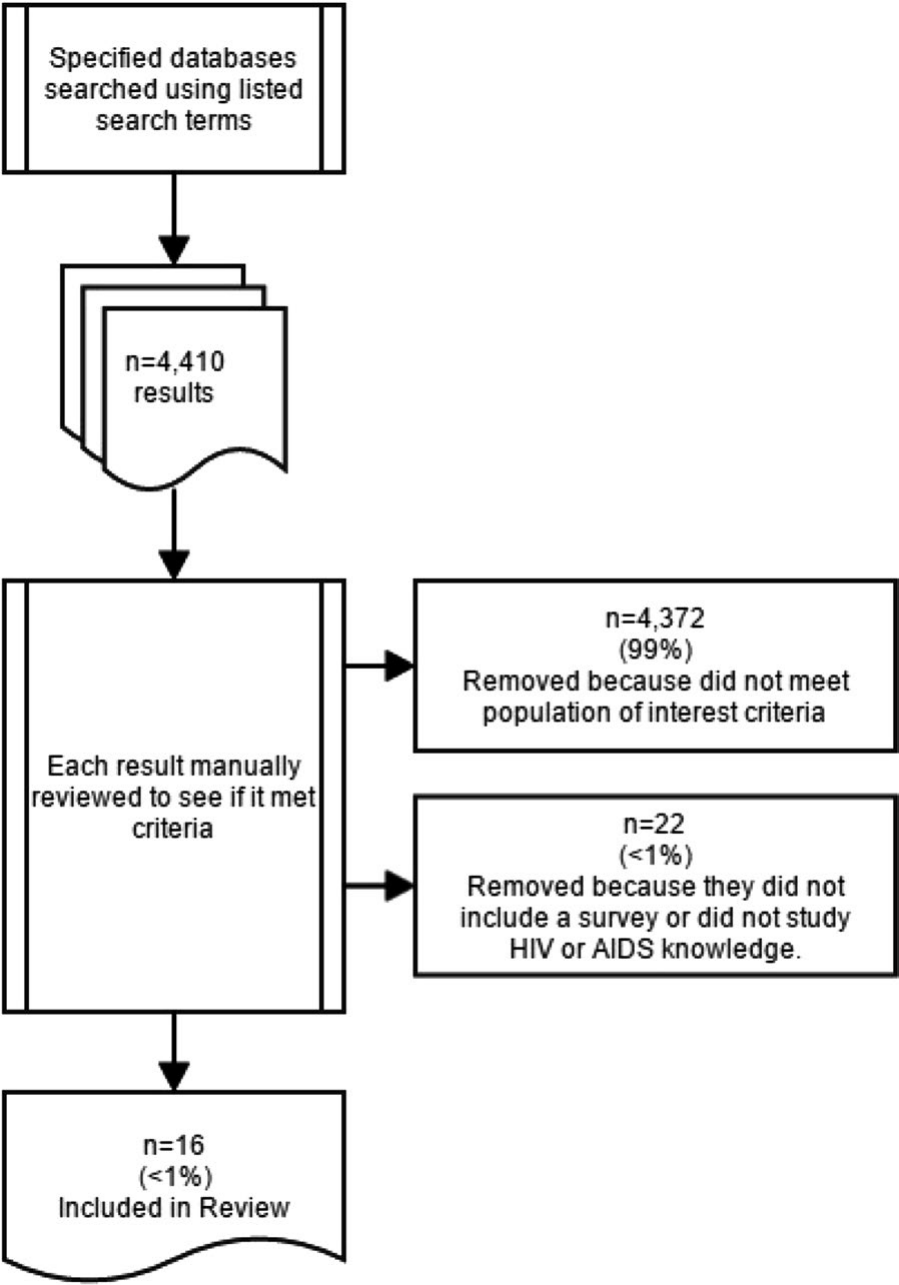
Article Flow Chart

The initial search for articles identified 4410 non-duplicated results (see Fig. 1). This included results from the databases searched that were listed. The abstracts of all 4410 results were reviewed manually to determine whether they met criteria. Of all these, n = 4372 (99 %) studies did not qualify because they did not concern the population of interest. A smaller percentage of studies (n = 22, <1 %) were disqualified because they did not include a survey, or they did not study HIV or AIDS knowledge. Ultimately, 16 articles met the criteria for this study (see Additional file 1: Table S1). No additional articles were identified by reviewing the reference list of qualifying articles. Also, none of the corresponding authors contacted to provide information responded to our request.

Additional file 1: Table S1 lists characteristics of the 16 studies in chronological order of publication that met the inclusion and exclusion criteria. Of these, 10 (63 %) were conducted in a KSA population [1, 2, 11, 13, 16, 17, 19, 20, 23, 28], and the rest were in neighboring countries (Kuwait n = 2 [22, 26], Yemen n = 2 [24, 25], UAE n = 1 [21], Oman n = 1 [29], Qatar = 0, Bahrain = 0). A majority of the articles studied students of primary, secondary, or post-secondary schools (n = 9, 56 %) [1, 2, 16, 17, 19, 21, 25, 27, 29], while others studied certain occupational cohorts, such as physicians [11, 22] and bus drivers [18] (n = 3) and others studied the general population (n = 3) [23, 24, 26]. Five studies included only men [1, 14, 16–18], while the other 11 included both sexes. Only one study was done on a clinical population [28]. A response rate was either unavailable or not reported for over half the studies (n = 9, 56 %) [2, 13, 17–19, 22, 26, 28, 29], but all studies that did report a response rate reported rates of at least 80 %, and three studies even achieved 100 % [1, 16, 25]. A variety of sampling approaches were used, the most common being some type of cluster sampling (n = 6, 38 %) [1, 13, 19, 22, 25, 28]. Sampling approaches were not reported for three studies [14, 17, 29].

Information about the measurement instruments used in these studies was unavailable or not reported for four studies (25 %) [13, 16, 17, 23], and the language of the questionnaire was unavailable or not reported for seven studies (44 %) [2, 17, 20–23, 28]. Six studies (38 %) [19, 21, 24–26, 29] used a measurement instrument based on a questionnaire developed by a health agency, such as the World Health Organization (WHO) Knowledge, Attitudes, Beliefs, and Practices (KABP) survey [30]. Two employed a questionnaire previously used in non-Arabic populations [1, 18], and two developed their own questionnaire for the study [14, 28]. Eight studies (50 %) reported administering the questionnaire in Arabic only [1, 13, 16, 19, 24–26, 29]. One study translated it into multiple languages to accommodate its multi-lingual population [18]. Information about the language of the questionnaire was unavailable or not reported for seven studies (44 %) [14, 17, 20–23, 28].

Of the 16 studies, only one commented on validity studies [22], and two commented on reliabilities studies [1, 22]. However, descriptions of reliability and validity studies as reported in the articles were not clear, in that they did not provide a description of how reliability or validity were assessed or evaluated in the study or previous studies.

This review found 16 HIV/AIDS knowledge questionnaires that could inform a current study of the KSA population. The main strengths identified across these studies included: careful approaches to sampling, adequate sample sizes, appropriate language of the questionnaire (when it was reported), and high response rates. The main weaknesses in the studies had to do with choice of population and choice of questionnaire, in addition to incomplete or confusing reporting on results.

As stated before, the content of the questionnaires was not reviewed for cultural-appropriateness, but the use of the instrument on an appropriate population was reviewed. Indeed, the most important weakness identified in the studies reviewed was the appropriateness of the population subject to the HIV or AIDS knowledge questionnaire. Given the culture of the KSA as described earlier, it is unclear as to why over half the studies reviewed (n = 9, 56 %) surveyed students, as this would likely constitute a low risk population for sexual transmission of HIV [1, 13, 14, 16, 17, 19, 21, 25, 29]. Indeed, studies show that HIV is more likely to be diagnosed in persons in the KSA over the age of 24 [13], and 60 % of cases are diagnosed in individuals aged 20–49 [5]. Therefore, the fact that over half of these 16 studies focused on younger populations means that HIV and AIDS knowledge is being measured in a low-risk population that likely would not benefit immediately from any public health intervention. Although it is important not to ignore a low-risk population in a public health effort, the first response of the public health system should be to intervene on those at high risk for the condition so as to hopefully prevent more cases. Hence, focusing on developing instruments to measure HIV knowledge in high risk populations is priority.

In KSA, because of its conservative approach to Islam, all public environments are gender-segregated as much as possible. For example, women and men use gender-specific banks. For this reason, it is surprising that no STI knowledge instrument targeting women or men only with respect to HIV and AIDS prevention knowledge has been developed in the time span of the studies reviewed. In fact, 5 of the 16 studies reviewed did not even include women, and no attempt was made to create gender-specific instruments when surveying men or mixed groups. As described earlier, KSA men are more likely to be infected by sex workers, and KSA women are more likely to be infected by their husbands. Therefore, HIV and AIDS knowledge questionnaires should be focused on the necessary knowledge to prevent transmission through these vectors.

Even though KSA follows a conservative approach to Islam, discussion of married women’s sexual behaviour is acceptable within a medical context, so participation in research is not compromised in this population. For example, a study on Saudi Arabian women’s experiences with maternal health services elicited very intimate comments from Saudi women about their pregnancy and birthing process [31].

In addition to concerns over the appropriateness of particular surveyed populations, criticism can be leveraged regarding the choice of instruments used in these studies. It is reasonable that six studies selected questionnaires developed by health agencies, but two studies used questionnaires based on non-Arabic populations, two used questionnaires that the authors developed for the study (and therefore, they had not been tested or reported on previously), and information about the questionnaire used was not available for four studies. This means that in 50 % of the studies reviewed, the questionnaire was likely to have a low level of accuracy. None of the instruments used provided the results of reliability or validity studies, so evaluating their quality is difficult.

Unfortunately, most of the HIV and AIDS knowledge instruments developed by health agencies, such as the WHO-KABP, are inappropriate for even high-risk populations in the KSA. The WHO-KABP was used in four studies and includes questions about HIV and AIDS pertinent to transmission by intravenous drug use (IDU) and homosexual activity [24–26, 29]. These questions are inappropriate for the KSA’s population, given its low HIV prevalence, low rates of IDU and homosexuality, and KSA’s strict expression of Islam. In the 2012 United National General Assembly Special Session on AIDS (UNGASS) country progress report [32], the KSA listed that the prevalence of HIV ranged between 0.03 % in lower risk groups to between 0.15 and 0.8 % for higher risk groups. Compare this to the US, where there are 50,000 new infections per year [33], or sub-Saharan African countries, who report that between 4 and 10 % of the general population is living with HIV with particular high-risk populations ranging as high as 20 % [34]. Rates of IDU have been reported to be higher in Jeddah but low overall in the KSA [10], and, “the estimated prevalence of heroin and amphetamine abuse in Saudi Arabia in 2000 as a percentage of the population aged 15 and above was 0.01 and 0.002 %, respectively” [5]. Homosexuality is banned by religion in KSA, and among homosexuals in KSA, the rates of HIV are low. This is confirmed by reports that among Saudis infected with HIV, <5 % of the infections came through IDU or homosexual activity [5].

While the instruments reviewed can be criticized for their choice of basing their instruments on Western ones that would be culturally-inappropriate for an Islamic population, their strengths should also be noted. Although it was impossible due to the lack of information provided to review the question content of instruments, some articles provided insight. One study reviewed contained a measurement instrument with the overarching question, “Can AIDS be transmitted through:” and included a series of 21 transmission routes where the respondent is asked to answer “yes” or “no” according to his/her knowledge [26]. Of these 21, the following 9 seem especially pertinent for high risk KSA populations [26] such as maternal [35] and heterosexual [36] transmission. Fageeh’s 2008 study included a more thoughtfully-developed measurement instrument that yielded more pertinent results [23]. In this study, a standard form titled “Awareness of Saudi Local Population on STDs” was used for data collection, which is described in the article as “a 5-part, 20 question questionnaire eliciting information about the knowledge of STDs” [23]. Direct, pertinent questions are asked, such as “Do you know how to protect yourself from STDs?” and “If you get an STD, do you think your partner is entitled to know?” Another question asked what actions the respondent would take upon finding out his/her partner had an STD (answer choices included: do nothing, I don’t know, get a check-up, ask him/her to get treated, avoid sexual contact, ask for divorce) [23]. A more recent study reviewed also included a survey with direct questions that focus on transmission routes common in this population (heterosexual transmission, mother-to-fetus, etc.), even though the instrument Fageeh uses is not mentioned as the basis for development [16]. Although the questions used in the more recent article’s survey, as well as Fageeh’s 2008 study, are more appropriate than those used in previous studies, question wording could be improved (e.g., to ask what types of protection the respondent knows about, not only whether s/he knows about protection), leaving challenges for the thoughtful development of a public health response [16, 23].

In addition to challenges with population selection and data collection instruments, the studies reviewed could also be criticized for the quality of their reporting. Many lacked a clear description of how the instruments were developed, how questions were selected, and how reliability and validity were ensured. These drawbacks severely hindered their comparison in terms of quality. One survey did not even describe the actual questions asked, making the article of limited value in terms of comparing HIV knowledge instruments [19]. In addition, many studies did not report basic information about survey development. Finally, no clear discussion was given to the public health response that would theoretically result from the answers to the questions.

One can imagine that the KSA Ministry of Health (MoH) would like to provide epidemiologically and culturally-appropriate HIV and AIDS prevention education to its high-risk populations using accurate statistics able to measure current attitudes and knowledge related to HIV and AIDS prevention. It will be difficult for the KSA to mount a public health response that is effective without gender-specific and culturally-appropriate instruments for assessing HIV and AIDS knowledge given the particular cultural features of KSA. Currently, no STI knowledge measurement instrument can be recommended for use in KSA men or women. In order to develop a culturally-competent quantitative measurement STI knowledge measurement instrument for married Saudi women, qualitative studies will need to be done that interview married Saudi women and gain a better understanding of both their level of knowledge as well as their knowledge demands.

## Conclusions

In summary, 16 papers were reviewed that included HIV knowledge measurement in the KSA or bordering populations. On the positive side, all studies report high response rates, but other features of these reports indicate challenges. First, the absence of a culturally-specific KSA instrument has resulted in the inability to reliably and accurately measure HIV knowledge in KSA populations. Next, studies examined employed a variety of instruments, but they generally did not report reliability and validity studies, so their relative quality could not be compared. Further, gender-specific instruments were not developed, although transmission patterns in the KSA suggest that transmission modes are potentially much more gender-specific than in other countries. Finally, these articles generally reported on low-risk populations in the KSA. The KSA’s public health goals should more specifically focus on measuring and improving knowledge in high-risk populations such as married women—an option currently limited by commonly available measurement instruments.

In fact, it is interesting to observe that those measuring knowledge, attitudes, and behavior surrounding HIV in the KSA seem disconnected from the other researchers in the KSA studying the epidemiology of these conditions. It is recommended that researchers working on the epidemiology of HIV infection in the KSA and those surveying the public seeking to inform an effective response work together to develop culturally-appropriate instruments that measure knowledge pertinent to KSA’s population. Had researchers such as Abolfotouh, Mahfouz, Madani, and Fageeh collaborated, despite their disparate geographic locations, they might have prevented the use of inappropriate questions from the WHO KABP and other US- or European-based questionnaires that include many questions irrelevant to the KSA population.

In the 20 years since the landmark paper in 1995 by Abolfotouh, the evolution towards reliable, valid, and pertinent and gender-specific instruments for measuring HIV knowledge in the KSA population did not take place. Hence, today, little is known about the level of knowledge about HIV prevention in high-risk groups in KSA. KSA-specific instruments measuring knowledge pertinent to the epidemiology of HIV are desperately needed to facilitate the development of an effective public health response capable of accurately reaching at-risk populations.

## Methods

To select appropriate studies and instruments about HIV/AIDS knowledge to review, the following inclusion and exclusion criteria were applied:

### Inclusion criteria

1. Must include discussion of a quantitative survey instrument used.
2. Quantitative survey must be given either as an interview or self-reported questionnaire.
3. Survey must include questions about knowledge HIV or AIDS in specific, but can include questions about other constructs (e.g., attitudes), and can include questions about other STIs.
4. Survey must be given in either English or Arabic, but can be given in additional languages.
5. Survey must be done in a population in the KSA or other countries bordering the KSA on the Arabian Peninsula, including: Kuwait, Oman, United Arab Emirates (UAE), and Qatar.
6. Article must be written in English.

### Exclusion criteria

There were no exclusion criteria. It was chosen not to exclude articles on instruments used on younger, unmarried populations because it was felt that reviewing these articles would inform the use of these instruments in older populations.

### Search strategy

Google Scholar and Google Web were utilized to search for and identify articles in all of the following databases: PubMed, PLoS, WHO publications, UN publications, news, and other peer-reviewed publication databases. The first author (MA) also conducted a search of the Google Web in Arabic to identify articles that met criteria, but no additional articles were found. Search terms used include the following: Saudi Arabia, HIV, “HIV knowledge”, questionnaire, instrument, survey, AIDS. Because HIV was identified in the scientific literature beginning in the 19^th^ century, no date restrictions were put on the search. Although articles focused on populations from all the countries on the Arabian Peninsula would meet criteria for the study, only Saudi Arabia was used as a search term. This is because it was discovered that adding other countries (Qatar, Yemen, etc.) to the search string did not identify additional articles.

### Data collection

Researchers manually read abstracts for search results to determine if articles met the study criteria listed above. If it was unclear, the full article was read to assess whether it met criteria. The reference list for every article that met some or all criteria was reviewed for articles that may have been missed in the general search, and those were reviewed for meeting criteria. No articles were found using this approach, as all had been identified previously in the search. This process was repeated until all articles likely to meet criteria had been manually reviewed.

Articles that met the study criteria were then selected. The primary author and the year of publication were noted. The population studied was described (in terms of gender as well as other characteristics), as well as the total population completing the instrument, and the response rate (if reported). The sampling method was also recorded, as well as a description of the questionnaire used. Finally, the language of the questionnaire, and any accompanying reliability or validity studies were noted and reported. If any of this information was not reported in the article, the corresponding author was contacted to obtain the missing information.

According to the original study design, the following parameters were to be collected from these 16 articles: type of scales used (single-item or multi-item), length of instrument (both in terms of number of questions and time taken to complete), and survey domains (e.g., transmission vector knowledge). However, the articles were often very short and often incomplete. Most gave little or no detail on the content of their measurement instruments. Therefore, this data were not collected as part of the study.

## Additional file

10.1186/s13104-015-1614-x Articles on HIV/AIDS knowledge and attitudes questionnaires reviewed.

## Abbreviations

AIDS: acquired immune deficiency syndrome
HIV: human immunodeficiency virus
KABP: knowledge, attitudes, beliefs and practices
KSA: Kingdom of Saudi Arabia
MoH: Ministry of health
STD: sexually transmitted disease
STI: sexually transmitted infection
UAE: United Arab Emirates
UNGASS: United Nations General Assembly Special Session
US: United States
WHO: World Health Organization
